# A review of papillary breast carcinoma in women attending a breast imaging centre in Johannesburg

**DOI:** 10.4102/sajr.v29i1.3092

**Published:** 2025-05-02

**Authors:** Musawenkosi M. Mthombeni, Nasreen Mahomed, Grace Rubin, Sharadini K. Gounden

**Affiliations:** 1 Department of Radiology, Faculty of Health Sciences, University of the Witwatersrand, Johannesburg, South Africa; 2 Department of Radiology, Faculty of Health Sciences, University of KwaZulu-Natal, Durban, South Africa

**Keywords:** radiology, breast imaging, breast cancer, papillary breast cancer, mammography, ultrasound, complementary modalities

## Abstract

**Background:**

Breast cancer ranks globally as the most prevalent cause of female deaths. Papillary breast carcinoma (PBC), a rare subtype of breast cancer, presents distinct challenges in diagnosis and management because of its unique histopathological features.

**Objectives:**

This study aims to determine the prevalence and main imaging findings of PBC in women attending a tertiary breast imaging centre.

**Method:**

A retrospective review of mammography and ultrasound imaging findings of female patients with histologically proven PBC, referred to a tertiary breast imaging centre over a 5-year period, was conducted.

**Results:**

The study included 102 female patients with a mean age of 53.8. Mammography detected masses in 93.02%, with calcifications in 41.2% and abnormal borders in 56.8%. Architectural distortion and asymmetry occurred in 27.5% and 28.4% respectively, both showing moderate correlation with PBC (*r* = 0.50, *p* = 0.009; *r* = 0.51, *p* = 0.0057). Ultrasound findings indicated irregular mass shapes (mean = 1.53), with hypoechoic patterns significantly associated with PBC (*r* = 0.40, *p* = 0.0013). Correlation analysis revealed strong associations between PBC and breast pain (*r* = 0.74, *p* < 0.0001), and erythema (*r* = 0.62, *p* < 0.0001). There was no significant association between the mammography and ultrasound findings (*p* = 0.495).

**Conclusion:**

The findings underscore the value of using mammography and ultrasound in the diagnosis of PBC, as the two modalities offer complementary information.

**Contribution:**

There is a paucity of data on the radiological findings of PBC in Africa. The current study prevalence mirrors global trends, highlighting the importance of ongoing surveillance and diagnostic accuracy.

## Introduction

Cancer stands as the second leading global cause of mortality, with approximately 10 million deaths attributed to the disease in 2020. The majority of this burden falls on low- and middle-income countries, accounting for 70% of the nearly 10 million cancer-related deaths worldwide in the same year. In sub-Saharan Africa, cancer incidence is anticipated to surge by over 92% between the years 2020 and 2040.^1^

Breast cancer has become the most commonly diagnosed cancer; it is reported to be responsible for about 2.3 million cases in 2020^2^ and is projected to reach about 4.4 million cases in 2070.^3^ In 2024, it is estimated that 42 250 women and 530 men in the United States will die of breast cancer.^4^ It is predominantely found in women and rarely diagnosed in men, though the prevalence in men has increased in recent years and sits at less than 1% of all breast cancer diagnoses.^5^ Among women in South Africa, breast cancer stands out as the most prevalent form of cancer, representing 23.22% of all newly diagnosed cancer cases in this demographic.^6^ Breast cancer is a significant health problem affecting the women who seek care. South Africa currently lacks a national breast cancer screening programme and standardised guidelines for breast cancer screening, unlike many high-income countries. This absence of a systematic approach may contribute to delays in diagnosis and treatment, often leading to advanced-stage presentations at diagnosis.^2,3^ The lack of structured screening and early detection initiatives highlights the need for tailored strategies to address breast cancer within the unique resource constraints of the healthcare system.

Breast cancer is a complex disease with various subtypes. Cancer develops within the lobules of the ducts of the breast and may emerge within the fatty and fibrous connective tissue present in the breast. The different types of breast cancer can be categorised based on where they originate and how they behave.^7^ The primary categories are non-invasive, in-situ breast cancers and invasive breast cancers. The latter category includes invasive ductal carcinomas, invasive lobular carcinomas, triple-negative breast cancer, her2-positive breast cancer, ER-positive and inflammatory breast cancer.^8^

Papillary breast carcinoma (PBC) is a rare subtype of breast cancer that accounts for only 1% – 2% of all breast cancer cases. It is characterised by the presence of papillary structures within the tumour tissue, resembling finger-like projections. It is more frequently diagnosed in postmenopausal women, but cases can occur in women of all ages.^9^ Papillary carcinomas are classified based on the appearance of their epithelium. When the epithelium resembles intraductal carcinoma, the tumour is termed papillary ductal carcinoma in situ. If there is a cystic component, it is then referred to as intracystic papillary carcinoma (ICPC). Without a noticeable cyst, it is termed solid papillary carcinoma. Invasive elements in papillary carcinomas are typically found at the lesion’s periphery.^8^

On clinical examination, it presents as a painless palpable mass with or without a nipple discharge.^4^ Given its distinct pathology, accurate preoperative diagnosis through advanced imaging techniques is crucial for optimising treatment strategies and improving patient outcomes. When there is a specific concern related to nipple discharge or to investigate issues within the breast ducts, galactography can be used but is not typically used as a primary method for the analysis of PBC.

Mammography remains the cornerstone in breast cancer detection, and its role in diagnosing PBC is no exception. The mammographic features of papillary lesions include the identification of suspicious microcalcifications, architectural distortions and lesion borders. The emphasis is placed on differentiating papillary lesions from other benign and malignant breast abnormalities.^10^ On mammography, the features of PBC may include a mass that appears distinct with defined or ill-defined margins, round or oval in shape. Calcifications within the lesion can vary in appearance and distribution. Ductal changes can appear as dilated ducts or distortion of the ductal architecture. There may be asymmetry on mammography, and microcalcifications which may appear clustered or dispersed within the lesion.^5^

Sonographic evaluation is integral in the diagnostic workup of PBC, offering real-time imaging and improved characterisation of lesions. Sonographic features associated with papillary lesions include the presence of intracystic papillary projections, active vascularity patterns and irregular masses. They also require differentiation of benign from malignant lesions.^11^ Features that may be seen on ultrasound for PBC include; a solid mass, but can also involve complex features with cystic areas or areas of necrosis within the lesion. The lesion may also demonstrate ill-defined borders demonstrating areas of infiltration into surrounding tissue. Papillary breast carcinoma may appear hypoechoic or isoechoic compared to surrounding breast tissue. Malignant lesions may show micro-lobulated or angular margins.^8^ Depending on the composition of the mass, posterior acoustic shadowing may or may not be present. Axillary lymphadenopathy may demonstrate abnormal morphology and increased vascularity on ultrasound.

The use of MRI in breast imaging provides a comprehensive assessment of lesion morphology and vascularisation. MRI enables sensitive detection of multifocal or multicentric disease, compared to other imaging modalities, necessary for surgical planning and response to treatment. Papillary breast carcinoma often presents with heterogeneous enhancement, reflecting the complex nature of the lesion. There will be washout kinetic curve characteristics of malignant lesions. Papillary breast carcinomas appear hyperintense on T2-weighted images because of their cellular composition and fluid content.^12^

An essential aspect of this study involves the integration of findings from different imaging modalities to enhance diagnostic accuracy. The synergistic interpretation of mammography and ultrasound results will be explored, highlighting the complementary nature of these techniques in characterising PBC. This multimodal approach aims to improve preoperative diagnostic certainty, allowing for more informed decision-making regarding treatment options.^13,14^

This study is unique within South Africa and sub-Saharan Africa, as few studies have comprehensively analysed PBC using multiple imaging modalities in this region. By examining the diagnostic value of both mammography and ultrasound for PBC, the findings contribute essential insights to the existing literature on breast cancer diagnostics in our setting. The study’s results could also serve as a foundation for developing national screening guidelines, potentially supporting more accurate diagnosis and improved outcomes for breast cancer patients in South Africa.

## Research methods and design

The study was a retrospective review of imaging findings of female patients, aged 18 years and older, with core biopsy histologically confirmed PBC. The study population included all patients referred to a tertiary hospital breast imaging unit between January 2017 and December 2023. Patients with a previous history of any malignancy and records with missing imaging findings were excluded. Given the rare nature of PBC, only a few cases were observed annually.

Patient files and clinical records were obtained through the hospital online database and the National Health Laboratory Services (NHLS). Clinical records that were assessed included tumour size, lymph node involvement and histological grade. The NHLS data were accessed, and specimen reference numbers were used to access the histology results. Data were initially screened for inconsistencies and missing values.

Descriptive statistics, including means, medians, frequencies and standard deviations, were used to summarise participant demographics and clinical characteristics. Hypothesis testing and significance level for each analysis, hypotheses were formulated, and a significance level (α) of 0.05 was set.

The following statistical tests were chosen based on the type of data and research objectives. For comparisons of continuous variables, *t*-tests were utilised. Chi-square tests were employed for analyses involving categorical variables. *p*-values were calculated to ascertain the significance of the results. Based on the data collected, the selected tests were deemed appropriate for the study’s objectives. Additionally, confirmed cases were analysed against presenting complaints, imaging findings, demographic data and other relevant variables. The statistical analysis indicated which variable made the highest contribution to a confirmed diagnosis. This approach further enhanced the understanding of the diagnostic process and helped identify key factors associated with PBC. Before each test, underlying assumptions were checked.

Data analysis was conducted using Minitab, incorporating descriptive statistics, normality assessment via the Anderson–Darling test, correlation analysis with heatmap visualisation and hypothesis testing using the Mann–Whitney *U* test for independent group comparisons. Given that most variables did not follow a normal distribution (*p* < 0.05), non-parametric tests were employed to ensure robust statistical evaluation. The results, including *p*-values and effect sizes, were transparently reported in tables and figures, with a focus on clear interpretation in the context of the study’s objectives.

### Ethical considerations

Ethical clearance to conduct this study was obtained from the University of the Witwatersrand, Human Research Ethics Committee (No. M240976).

## Results

From 1784 total records of confirmed cancer cases, there were a total of 102 (0.05%) female patients included with a histologically confirmed diagnosis of PBC on core biopsy. The age distribution of the participants, ranged from 24 to 89 years; the median age was 54 years, with an interquartile range of 43 to 63.5 years. Descriptive statistics are summarised in Table 1.

**TABLE 1:** Summary of clinical and imaging variables in the study population (*N* = 102).

Factor assessed	Variable	*n*	%	Mean
Age (years)		-	-	53.80
Clinical symptoms	Palpable mass	81	79.4	0.90
	Nipple discharge	52	50.9	0.02
	Breast Erythema	53	51.9	0.13
	Breast pain	52	50.9	0.15
	Swelling of the breast	53	51.9	0.13
Mammography	Mass	76	74.5	0.91
	Calcifications	42	41.2	1.61
	Abnormal borders	58	56.8	2.98
	Asymmetry	28	28.4	0.32
	Architectural distortion	26	27.5	0.30

**FIGURE 1:**
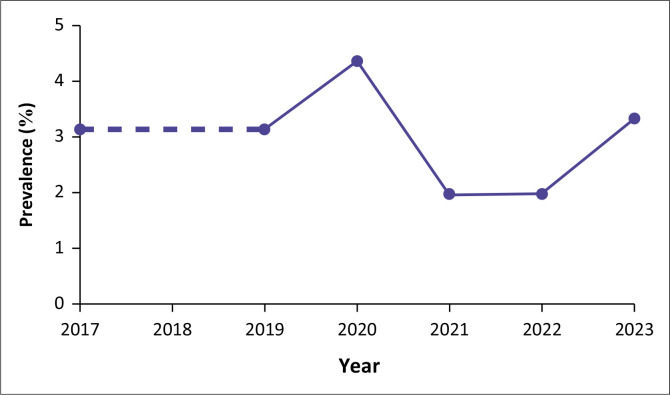
Prevalence of papillary breast carcinoma between 2017 and 2023.

The prevalence of PBC between 2017 and 2023 is demonstrated in Figure 1. There was no statistically significant trend in the prevalence of PBC over the years (*p* = 0.61).

### Imaging findings

Mammogram findings revealed significant differences in the imaging features between PBC and non-PBC cases. The analysis of masses showed a high prevalence with a mean value of 0.92, and the data significantly deviated from normality (*p* < 0.005). Similarly, the distribution of suspicious calcifications had a mean of 1.61, with non-normality confirmed (*p* < 0.005). For borders (circumscribed, indistinct, microlobulated, obscured, spiculated), a mean value of 2.63 was observed, with moderate variability, and a significant difference between PBC and non-PBC cases (*p* < 0.005).

Ultrasound findings revealed variations in the shapes of masses (irregular, oval or round), with the majority showing a consistent pattern around a mean value of 1.53. Despite some variability, the data strongly suggest a notable difference in mass shapes when comparing PBC to non-PBC cases (*p* < 0.005, CI 1.32 0 1.73).

For mammography, masses were detected in 93.02% of cases and not detected in 6.98%. Similarly, ultrasound detected masses in 91.07% of cases, with 8.93% showing no mass detection. A Chi-Square Test of Independence was performed to assess the association between mammography and ultrasound findings. The test yielded a Chi-Square statistic (χ^2^) of 0.467, with one degree of freedom and a *p*-value of 0.495. Given that the *p*-value exceeds the significance threshold of 0.05, we fail to reject the null hypothesis, indicating no significant association between mammography and ultrasound findings in detecting masses.

### Statistical correlations

**FIGURE 2:**
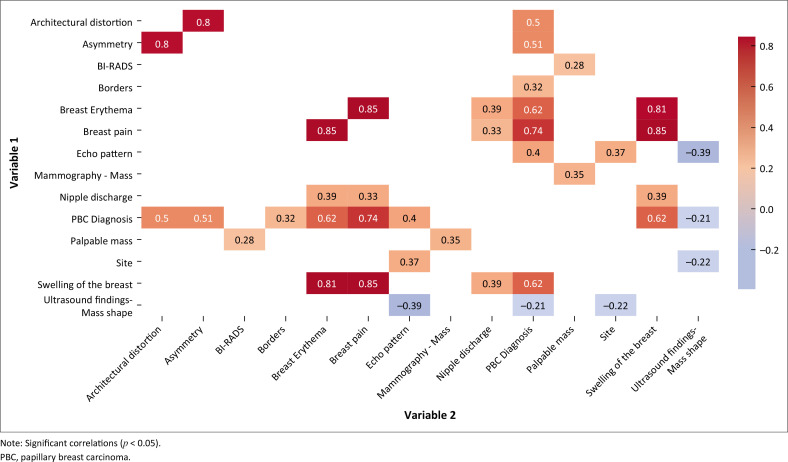
Heat map of correlation of variables and papillary breast carcinoma diagnosis.

The *p* values for each correlation were also calculated. The correlations for the relationships which are significant (*p* < 0.05) are shown in the simplified heat map (Figure 2). Darker red indicates a stronger positive correlation, and darker blue indicates a stronger negative correlation. These correlations indicate the strength and direction of the linear relationships between variables.

The analysis identified correlations between certain clinical and imaging findings and a PBC diagnosis. There was a strong positive correlation between breast erythema and PBC (*r* = 0.6248, *p* < 0.0001) as well as between breast pain and PBC (*r* = 0.7386, *p* < 0.0001), indicating these symptoms are highly associated with a PBC diagnosis. Moderate correlations were also noted for asymmetry (*r* = 0.5088, *p* = 0.0057), architectural distortion (*r* = 0.5007, *p* = 0.0092) and abnormal ultrasound echo pattern (*r* = 0.4042, *p* = 0.0009), suggesting these imaging features may also relate to PBC. However, a significant negative correlation was found between mass shape on ultrasound and PBC diagnosis (*r* = −0.2098, *p* = 0.0392), indicating that a particular mass shape may be less indicative of PBC.

Focusing on the variables that have a strong correlation with a PBC diagnosis, hypothesis testing was conducted to determine the relationships that were statistically significant (Table 2).

**TABLE 2:** Hypothesis testing of variables strongly correlated with papillary breast carcinoma diagnosis.

Variable	*p*
Breast pain	0.000000147
Breast Erythema	0.000007221
Swelling of the breast	0.000007221
Abnormal ultrasound echo pattern	0.001297461
Ultrasound Mass shape	0.006562498
Mammography asymmetry	0.008968052
Mammography architectural distortion	0.013491320
Mammography borders	0.041392710
Nipple discharge	0.063036543
*p*-value target	0.050000000

Clinically, features such as breast erythema, breast pain and swelling of the breast, along with radiological findings like ultrasound mass shape, abnormal ultrasound echo patterns and mammographic architectural distortion, were strongly associated with a PBC diagnosis. Though nipple discharge may indicate PBC diagnosis and was present in 50.9% of cases, the *p*-value was significantly higher than the statistical threshold of significance in this data set. Breast pain has the most significant difference, as indicated by the smallest *p*-value and largest *t*-statistic.

Mammography and ultrasound demonstrated a high rate of mass detection, with over 90% usefulness for both modalities. However, the Chi-Square test result (*p*-value = 0.495) showed no significant association between the findings of mammography and ultrasound, suggesting that the detection of a mass by one method does not necessarily influence the detection by the other. A weak positive correlation (*r* = 0.28) was observed between the two methods, suggesting some level of agreement on mass detection. While modest, this correlation highlights the potential complementary value of combining the two imaging modalities in clinical practice.

The analysis of different mass types revealed key findings regarding the association between mammography and ultrasound (Table 3). For certain mass types, a significant relationship was observed between the findings of the two imaging modalities, indicating that they provide complementary information rather than independent results. Specifically, for nodules and mixed solid cystic – lobulated masses, the Chi-Square statistic of 20.988 and a *p*-value of 0.0001 (with three degrees of freedom) suggest a strong association, implying that the detection of these masses by both mammography and ultrasound is interdependent. This means that using both modalities together enhances the accuracy of diagnosing these specific mass types.

**TABLE 3:** Chi-square test results for each mass type.

Mass type	Chi-square statistic	*p*-value	*df*	Interpretation
Nodule	20.99	0.0001	3	Significant
Mixed solid cystic – lobulated	20.99	0.0001	3	Significant
Intraductal solid mass	0.13	0.9874	3	Not significant
Lobulated mass + smaller lesions	0.13	0.9874	3	Not significant

*df*, degrees of freedom.

In contrast, for intraductal solid masses and lobulated masses with smaller lesions, there was no significant association between the two methods (Chi-Square statistic of 0.135 and *p*-value of 0.9874), indicating that in these cases, the two modalities operate more independently.

**FIGURE 3:**
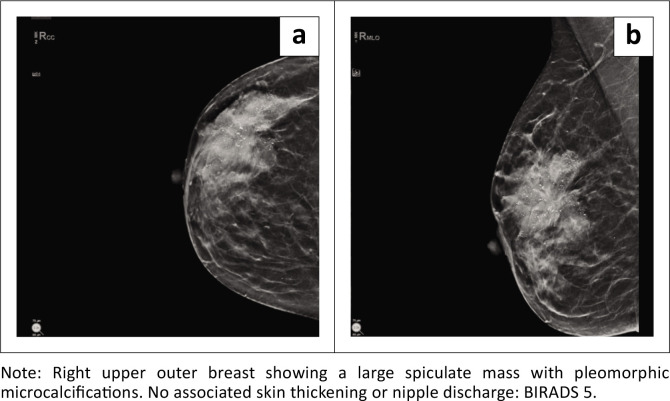
Mammographic findings: Suspicious dense mass with pleomorphic microcalcifications.

**FIGURE 4:**
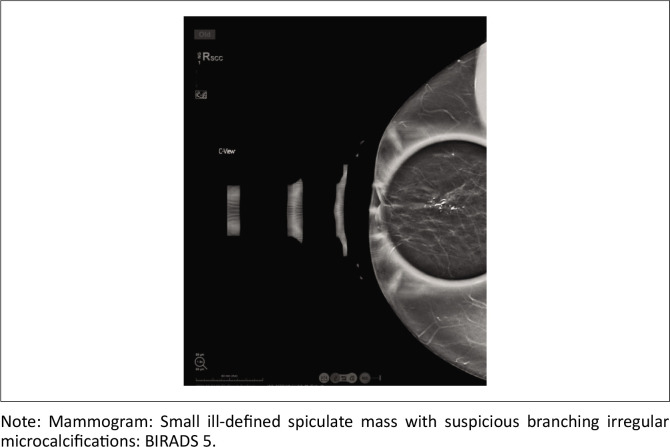
Mammographic findings: Branching calcifications with BIRADS 5 classification.

Mammography detected masses in 93.02% of cases, highlighting its high sensitivity for PBC-related abnormalities. Calcifications were present in 41.2% of cases, with suspicious patterns often associated with malignancy (Figure 3 and Figure 4). Abnormal borders (56.8%) and architectural distortion (27.5%) showed moderate correlations with PBC (*r* = 0.32, *p* = 0.041; *r* = 0.50, *p* = 0.009, respectively). Asymmetry was observed in 28.4% of cases and had a moderate correlation with PBC (*r* = 0.51, *p* = 0.0057). Ultrasound findings revealed that irregular mass shapes (mean = 1.53) were significantly associated with malignancy (*r* = −0.21, *p* = 0.039), while hypoechoic masses (mean = 1.92) showed a moderate positive correlation with PBC (*r* = 0.40, *p* = 0.0013). Breast pain (*r* = 0.74, *p* < 0.0001) and breast erythema (*r* = 0.62, *p* < 0.0001) were the strongest clinical predictors of PBC. These findings underscore the complementary role of mammography and ultrasound in PBC diagnosis, particularly in resource-limited settings.

## Discussion

Clinically, PBC typically presents as a palpable breast mass, often with associated symptoms like pain or nipple discharge. It tends to have a more favourable prognosis compared to other invasive breast cancers because of its slow-growing nature and lower likelihood of lymph node involvement. However, the challenge lies in accurately diagnosing PBC preoperatively, as its imaging characteristics can sometimes overlap with benign breast lesions such as fibroadenomas or papillomas.^8,11^

Radiologically, PBC can present as a well-circumscribed mass with or without calcifications on mammography. On ultrasound, it often appears as a solid mass with posterior acoustic enhancement, but these findings are not specific to PBC. The study’s analysis of mammography and ultrasound findings revealed that while both imaging modalities are effective at detecting masses, they operate relatively independently, with no significant statistical association between their findings (*p*-value = 0.495). This suggests that mammography and ultrasound provide complementary information in diagnosing PBC, which aligns with the global understanding of imaging practices for breast cancer.

Globally, mammography and ultrasound are the primary imaging techniques used to detect breast lesions. The scarcity of published data on PBC in LMICs, particularly within African and Asian contexts, underscores the significance of this study. In LMICs, breast cancer often presents at advanced stages, leading to higher mortality rates compared to high-income countries. This disparity is attributed to limited access to screening and diagnostic resources, as well as delayed treatment initiation.^15^ Given the constraints in LMICs, alternative diagnostic tools are essential. Ultrasound, for instance, has been identified as a valuable modality in resource-limited settings because of its affordability and effectiveness, especially when combined with clinical breast examinations.^16^

The study findings from the breast imaging centre in Johannesburg are consistent with existing literature, where mammography tends to have a higher sensitivity for detecting calcifications, while ultrasound is more sensitive in identifying solid masses. The weak correlation between these modalities, as found in this study, further supports the notion that both methods should be used in tandem to ensure a comprehensive evaluation. This is especially important in the case of PBC, where the diagnostic process can be challenging because of its subtle imaging features.

The lack of a significant association for other mass types also highlights the fact that each imaging modality may offer unique insights, highlighting the value of a multimodal approach in breast cancer diagnostics. The results highlighted the potential application of precision medicine in breast cancer diagnostics. By identifying specific clinical and imaging features strongly associated with PBC, such as breast erythema and pain, tailored diagnostic approaches can be developed. Therefore, leveraging personalised diagnostic tools to improve early detection and management.

The prevalence analysis over the period 2017–2023 showed no statistically significant trend in the occurrence of PBC, with a *p*-value of 0.61. This lack of trend is consistent with the global rarity of the disease and suggests that the prevalence of PBC in this South African cohort has remained stable over the years. Furthermore, the absence of MRI as a diagnostic tool in this study because of resource limitations highlights a significant disparity in healthcare access compared to more resource-rich settings where MRI is often used to evaluate complex breast lesions. Much of the existing literature on PBC originates from high-income countries, such as the United States and European nations, where healthcare systems typically have greater access to advanced diagnostic resources, including MRI. In South Africa’s public healthcare sector, limited access to MRI necessitates reliance on mammography and ultrasound for breast cancer diagnosis. This study highlights the effectiveness of these methods for characterising PBC, emphasising their critical role in resource-constrained settings.

This study was undertaken because of the rarity and potential aggressiveness of PBC, and although uncommon, can present with invasive characteristics and carry a significant risk of recurrence. Given its rarity, PBC is underrepresented in existing literature, particularly within African populations where data on diagnostic approaches and outcomes remain sparse. The age data, with a mean of 53.8 years, underscores the importance of mammograms for women over 40, as early detection in this age group is crucial. Establishing evidence-based guidelines for rare cancer types such as PBC is central to ensure timely and accurate diagnosis, which could significantly improve patient outcomes.

South Africa lacks a national breast cancer screening policy, with screening initiatives largely confined to private sector programmes. The absence of standardised national guidelines can contribute to delayed diagnoses, which is associated with more advanced disease at presentation and poorer outcomes. By providing data on the effectiveness of mammography and ultrasound for PBC diagnosis, this study contributes valuable insights to the existing literature and supports the need for context-specific screening guidelines. In the long term, the findings may aid in the development of national screening policies, enabling earlier detection and improved management of breast cancer in South Africa.

Limitations to the study included observer variability, as differences in radiologist interpretation may affect the consistency of imaging classifications and diagnoses. Variability in the application of the BI-RADS classification system could further influence diagnostic accuracy, particularly in distinguishing borderline cases. Additionally, inconsistencies in data capturing and record-keeping may have impacted the completeness and reliability of patient information, potentially introducing bias in the analysis.

## Conclusion

Breast cancer is the most prevalent cancer among South African women, yet studies of PBC are limited. This study provides insights into the diagnostic value of mammography and ultrasound for PBC in our local clinical setting, laying the groundwork for future screening guidelines in resource-limited settings. The findings of this study underscore the value of using both mammography and ultrasound in the diagnosis of PBC, with the two modalities offering complementary information. While there is no significant association between the two methods’ findings, their combined use remains crucial for comprehensive diagnosis, particularly in regions like South Africa where more advanced imaging modalities such as MRI may not be readily available. Furthermore, the stable prevalence of PBC observed in this cohort mirrors global trends, highlighting the importance of ongoing surveillance and diagnostic accuracy for this rare but significant breast cancer subtype.

## References

[1] WHO International Agency for Research on Cancer. Estimated number of deaths in 2020, all cancers, both sexes, all ages [homepage on the Internet]. Cancer Today; 2020 [cited 2024 Jan 16]. Available from: https://gco.iarc.fr/today/online-analysis-pie?v=2020&mode=population&mode_population=income&population=900&populations=900&key=total&sex=0&cancer=39&type=1&statistic=5&prevalence=0&population_group=0&ages_group%5B%5D=0&ages_group%5B%5D=17&nb_items=7&group_cancer=1&include_nmsc=1&include_nmsc_other=1&half_pie=0&donut=0

[2] Sung H, Ferlay J, Siegel RL, et al. Global cancer statistics 2020: GLOBOCAN estimates of incidence and mortality worldwide for 36 cancers in 185 countries. CA Cancer J Clin. 2021;71(3):209–249. 10.3322/caac.21660 33538338

[3] Soerjomataram I, Bray F. Planning for tomorrow: Global cancer incidence and the role of prevention 2020–2070. Nat Rev Clin Oncol. 2021;18(10):663–672. 10.1038/s41571-021-00514-z 34079102

[4] Siegel RL, Giaquinto AN, Jemal A. Cancer statistics, 2024. CA Cancer J Clin. 2024;74(1):12–49. 10.3322/caac.21820 38230766

[5] Momenimovahed Z, Salehiniya H. Epidemiological characteristics of and risk factors for breast cancer in the world. Breast Cancer (Dove Med Press). 2019; 11:151–164. 10.2147/BCTT.S176070 31040712 PMC6462164

[6] CANSA: Cancer Association of South Africa. Summary statistics of cancer diagnosed histologically in 2019 female – All population groups combined [homepage on the Internet]. 2019 [cited 2024 Jan 19]. Available from: https://cansa.org.za/files/2022/03/NCR_Path_2019_Full_Report_8dec2021.pdf

[7] Makki J. Diversity of breast carcinoma: Histological subtypes and clinical relevance. Clin Med Insights Pathol. 2015;8:CPath.S31563. 10.4137/CPath.S31563 PMC4689326 26740749

[8] Gannon LM, Cotter MB, Quinn CM. The classification of invasive carcinoma of the breast. Expert Rev Anticancer Ther. 2013;13(8):941–954. 10.1586/14737140.2013.820577 23984896

[9] Rakha EA, Ellis IO. Diagnostic challenges in papillary lesions of the breast. Pathology. 2018;50(1):100–110. 10.1016/j.pathol.2017.10.005 29179906

[10] Catanzariti F, Avendano D, Cicero G, et al. High-risk lesions of the breast: Concurrent diagnostic tools and management recommendations. Insights Imaging. 2021;12(1):63. 10.1186/s13244-021-01005-6 34037876 PMC8155169

[11] Gao L, Lai X, Zhang J, Jiang Y, Li J. Sonographic prediction of intraductal papillary carcinoma with partially cystic breast lesions. BMC Med Imaging. 2023;23(1):3. 10.1186/s12880-022-00934-y 36609236 PMC9817258

[12] Ali Salman R. Prevalence of women breast cancer. Cell Mol Biomed Rep. 2023;3(4):185–196. 10.55705/cmbr.2023.384467.1095

[13] Bray F, Ferlay J, Soerjomataram I, Siegel RL, Torre LA, Jemal A. Global cancer statistics 2018: GLOBOCAN estimates of incidence and mortality worldwide for 36 cancers in 185 countries. CA Cancer J Clin. 2018;68(6):394–424. 10.3322/caac.21492 30207593

[14] Kurtoğlu Özçağlayan Tİ, Öznur M. Digital mammography, ultrasound and magnetic resonance imaging characteristics in differential diagnosis of papillary carcinoma subtypes of the breast and diagnostic challenges. Eur J Breast Health. 2022;18(2):172–181. 10.4274/ejbh.galenos.2022.2021-9-4 35445176 PMC8987858

[15] Martei YM, Pace LE, Brock JE, Shulman LN. Breast cancer in low- and middle-income countries. Clin Lab Med. 2018;38(1):161–173. 10.1016/j.cll.2017.10.013 29412880 PMC6277976

[16] Dan Q, Zheng T, Liu L, Sun D, Chen Y. Ultrasound for breast cancer screening in resource-limited settings: Current practice and future directions. Cancers (Basel). 2023;15(7):2112. 10.3390/cancers15072112 37046773 PMC10093585

